# Functionalized Persistent Luminescence Nanoparticle-Based Magnetic Separation Aptasensor for Autofluorescence-Free Determination of *Salmonella enteritidis*

**DOI:** 10.3390/foods15081273

**Published:** 2026-04-08

**Authors:** Lixia Yan, Liufeng Yu, Ling Sun, Beibei Wang, Yi Zhang

**Affiliations:** 1 State Key Laboratory of Food Science and Resources, Jiangnan University, Wuxi 214122, China; yuliuf0204@163.com; 2 Instrumental Analysis and Laboratory Animal Center, Jiangnan University, Wuxi 214122, China; 3 School of Food Science and Technology, Jiangnan University, Wuxi 214122, China; 4 Institute for Egg Science and Technology, School of Food and Biological Engineering, Chengdu University, Chengdu 610106, China; 212023095135056@cdu.edu.cn (L.S.); wangbeibei@cdu.edu.cn (B.W.); 5 Food Safety Detection Key Laboratory of Sichuan Province, Chengdu 610000, China

**Keywords:** persistent luminescence nanoparticle, *Salmonella enteritidis*, aptasensor, magnetic separation

## Abstract

*Salmonella enteritidis* (SE) is recognized as a primary etiological agent of foodborne infection and food poisoning. Selective and sensitive determination of SE in animal-derived products is of great importance for ensuring safety in the food industry. Here, we report a highly sensitive and specific competition assay for detecting SE in eggs without interference from background fluorescence, by using persistent luminescent nanoparticles (PLNPs) as luminescent probes in combination with aptamer recognition and magnetic separation. Initially, the SE-specific aptamer (SEapt), as previously reported, was conjugated onto the surface of Fe_3_O_4_ magnetic nanoparticles to serve as both the recognition and separation unit. Meanwhile, the ZnGa_2_O_4_:Cr (PLNPs) were functionalized with the aptamer-complementary DNA (cDNA), serving as the PL signal generator. The constructed PL aptasensor is composed of the aptamer-conjugated MNPs (MNPs-SEapt) and cDNA-functionalized PLNPs (PLNPs-cDNA), integrating the merits of the long-lasting luminescence of PLNPs, the magnetic separation ability of MNPs and the selectivity of the aptamer. This integration offers a promising approach for autofluorescence-free determination of SE in food samples. The proposed aptasensor exhibited excellent linearity in the range from 1.0 × 10^2^–1.0 × 10^7^ CFU mL^−1^ with a limit of detection as low as 32 CFU mL^−1^. The precision for 11 replicate determinations of 1.0 × 10^3^ CFU mL^−1^ SE was 3.4% (relative standard deviation). The developed aptasensor achieved recoveries ranging from 98.8% to 102.8% for the determination of SE in the presence of common foodborne bacterial interferents. The method was successfully applied to the analysis of Salmonella genus in egg samples. In principle, the proposed platform may be adapted to other food matrices by substituting the target-specific aptamer, pending target-dependent optimization and validation.

## 1. Introduction

*Salmonella enteritidis* (SE) primarily causes gastroenteritis in livestock and poultry, as well as human enteritis and foodborne illness, and is recognized as a major etiological agent of foodborne infection and food poisoning [1,2]. The predominant route of human infection is the consumption of SE-contaminated foods, particularly animal-derived products such as meat, eggs, and milk [3]. Worldwide, SE ranks first or second among the causative agents implicated in food poisoning outbreaks, which has attracted substantial public and scientific attention [4]. Therefore, the development of early bacterial identification technologies is crucial for ensuring safety in the food industry and for clinical diagnosis. At present, the detection of SE is mainly based on conventional culture-based isolation methods [5], enzyme-linked immunosorbent assays [6], and polymerase chain reaction [7]. However, these approaches generally do not enable real-time monitoring of samples and, to varying degrees, suffer from labor-intensive and time-consuming procedures, as well as complex sample pretreatment. Consequently, an urgent need to establish a simple, rapid, and highly sensitive method for SE detection is essential.

Fluorescence-based methods are widely favored owing to their high sensitivity, simple design, and methodological flexibility [8,9,10]. However, most fluorescent probes require continuous excitation by a constant external light source during operation, which readily induces autofluorescence from the sample matrix and generates interfering signals [11]. Currently, strategies capable of suppressing autofluorescence mainly rely on X-ray-excited luminescent nanomaterials and persistent luminescent nanomaterials that do not require in situ excitation. Persistent luminescence refers to the phenomenon in which emission persists for minutes, hours, or even days after the excitation is stopped [12]. Persistent luminescence nanoparticles (PLNPs), also termed long-afterglow nanoparticles, can maintain an afterglow signal for a period of time after removal of the excitation light and thereby produce persistent luminescence (PL) [13,14]. This feature renders PLNPs particularly attractive for bioimaging and biosensing [15]. Food matrices are compositionally complex, typically containing lipids, starches, proteins, sugars, or vitamins, and under ultraviolet excitation, matrix components such as sugars and proteins may produce interfering fluorescent signals [16,17]. In addition, the residue levels of hazardous analytes are usually low. Therefore, highly sensitive analytical methods are required to meet detection demands. Using the afterglow emission of PLNPs as the sensing signal is expected to eliminate background fluorescence interference from food matrices and improve detection sensitivity and selectivity [18]. Nevertheless, the analytical application of PLNPs in food samples remains very limited.

The analysis and detection of foodborne pathogens involve analytical techniques for complex mixed samples. Selecting appropriate enrichment and separation approaches to isolate the target from complex food matrices while simultaneously eliminating background interference from the matrix is key to establishing accurate, rapid, and highly sensitive pathogen detection technologies [19]. In recent years, the development of magnetic nanomaterials has become increasingly mature; the synthesis of magnetic nanoparticles has been well established, and their performance has been continuously improved [20]. As a result, they have been widely applied to the enrichment and separation of foodborne pathogens. Under an external magnetic field, magnetic nanoparticles can enable rapid, efficient, and impurity-interference-reducing performance, potentially obviating the enrichment culture step. By integrating magnetic nanoparticles with various analytical methods, targets can be rapidly enriched from complex systems, which helps shorten the detection time and increase the detection rate of the target [21].

Herein, a highly sensitive and specific method for detecting SE in eggs without interference from background fluorescence was developed, by using PLNPs as luminescent probes in combination with aptamer recognition and magnetic separation. First, near-infrared-emissive PLNPs (ZnGa_2_O_4_:Cr) were synthesized as signal-reporting probes. The complementary DNA (cDNA) of the SE aptamer (SEapt) [22,23] was conjugated onto the surface of PLNPs to form a cDNA-functionalized signal probe (PLNPs-cDNA). Magnetic Fe_3_O_4_ nanoparticles were modified with the SEapt to serve as both the recognition and separation unit. The aptamer-modified Fe_3_O_4_ (Fe_3_O_4_-SEapt) was then hybridized with PLNPs-cDNA via complementary base pairing to construct a composite probe for SE detection. By optimizing the assay conditions, a highly sensitive detection method was established based on the previously reported selective SEapt [22,23], and its linear range, limit of detection, selectivity, and anti-interference performance in real samples were evaluated.

## 2. Materials and Methods

### 2.1. Chemicals and Materials

Unless stated otherwise, all reagents employed in this study were of analytical grade and utilized directly without any further purification. Gallium nitrate hydrate (Ga(NO_3_)_3_·XH_2_O, 99.9%), zinc nitrate hexahydrate (Zn(NO_3_)_2_·6H_2_O, 99%), chromium nitrate nonahydrate (Cr(NO_3_)_3_·9H_2_O, 99.99%), FeCl_3_·6H_2_O, 3-aminopropyltriethoxysilane, 1,6-hexanediamine, and 4-(2-hydroxyethyl)-1-piperazineethanesulfonic acid (HEPES) were obtained from Aladdin (Shanghai, China). Sulfo-N-succinimidyl 4-(maleimidomethyl) cyclohexane-1-carboxylate sodium salt (Sulfo-SMCC) was obtained from Macklin Biochemical Co., Ltd. (Shanghai, China). The *Salmonella enteritidis* (Order NO. A330788), sulfhydryl-modified Salmonella enteritidis aptamer and its partial complementary DNA (cDNA) (as detailed in Table 1) were obtained by Shanghai Sangon Biological Science & Technology Co., Ltd. (Shanghai, China). *Escherichia coli* (ATCC 25922), *Staphylococcus aureus* (ATCC 25923), and *Listeria monocytogenes* (ATCC 19115) were purchased from Guangdong Huankai Microbial Sci. & Tech. Co., Ltd. (Guangzhou, China). Wahaha ultrapure water (Hangzhou, China) was used for all experimental procedures. Phosphate-buffered saline (PBS) buffer (10 mM NaH_2_PO_4_-Na_2_HPO_4_, 100 mM NaCl, pH 7.4) served as the working buffer throughout the experiment.

### 2.2. Instrumentation

The dimensions and morphological features of the synthesized nanoparticles were examined using a JEM-2100 transmission electron microscope (TEM) manufactured by JEOL Corporation (Tokyo, Japan). A D2 PHASER powder diffractometer equipped with a Cu Kα radiation source (Bruker, Ettlingen, Germany) was employed to record the X-ray diffraction (XRD) profiles. UV–visible (UV-vis) absorption spectra were obtained with a UV-3600 PLUS spectrophotometer supplied by Shimadzu Corporation (Kyoto, Japan). The hydrodynamic particle size and zeta potential were determined via a Nano ZS Zeta sizer from Malvern Instruments (Malvern, UK). Photoluminescence (PL) measurements were performed in phosphorescence mode using an F-7000 fluorometer produced by Hitachi (Tokyo, Japan). Additionally, the magnetic characteristics of the samples were investigated with the PPMS-9 physical property measurement system (Quantum Design, San Diego, CA, USA).

### 2.3. Synthesis of Aptamer-Functionalized Fe_3_O_4_

Amino-functionalized Fe_3_O_4_ magnetic nanoparticles (Fe_3_O_4_-NH_2_) were synthesized according to the method reported by Wang et al. [24], and subsequently functionalized with SEapt to fabricate the capture probe. Specifically, 3 mg of Fe_3_O_4_-NH_2_ and 0.5 mg of sulfo-SMCC were dispersed in HEPES buffer (3 mL, 10 mM, pH 7.2), followed by ultrasonication for 5 min to ensure homogeneous dispersion. The mixture was shaken at room temperature for 2 h to activate the amino groups on the magnetic nanoparticles. After magnetic separation, the maleimide-activated Fe_3_O_4_-NH_2_ was washed three times with ultrapure water, and then re-dispersed into 3 mL of PBS buffer. Subsequently, the aptamer (SEapt, 3 nmol) was added to the above Fe_3_O_4_-NH_2_ suspension, and the mixture was shaken at room temperature overnight to prepare the Fe_3_O_4_-SEapt capture probe. The resulting Fe_3_O_4_-SEapt was collected by magnetic separation and washed three times with PBS buffer to remove excess aptamer. Prior to each use, Fe_3_O_4_-SEapt was re-dispersed in PBS buffer.

### 2.4. Hydrothermal Synthesis of PLNPs and Amino Functionalization

ZnGa_2_O_4_:Cr was synthesized via a hydrothermal method. Briefly, 1.0 mmol of zinc nitrate and 2.0 mmol of gallium nitrate were dissolved in 15 mL of ultrapure water. Under vigorous stirring, 0.004 mmol of chromium nitrate solution was added. The pH of the mixed solution was adjusted to 8.0 using aqueous ammonia, and the mixture was stirred at room temperature for 1 h. The resulting precursor solution was transferred to a Teflon-lined stainless-steel autoclave and heated at 220 °C for 20 h. After the reaction, the supernatant in the autoclave was discarded, and the precipitate was collected. The product was centrifuged to remove unreacted ions, washed three times with ultrapure water and ethanol, vacuum-dried at 50 °C, and ground for further use.

The as-prepared PLNPs were amino-functionalized as follows. Firstly, 100 mg of PLNPs was ultrasonically dispersed in 100 mL of 5 mmol/L NaOH solution and vigorously stirred for 12 h to obtain hydroxylated PLNPs (PLNPs-OH). The PLNPs-OH were collected by centrifugation and vacuum-dried. Subsequently, 100 mg of PLNPs-OH was ultrasonically dispersed in 100 mL of anhydrous ethanol. Under magnetic stirring, 3-aminopropyltriethoxysilane (400 μL) was added dropwise, and the mixture was stirred at 60 °C for 12 h. The resulting amino-functionalized PLNPs (PLNPs-NH_2_) were collected by centrifugation, washed with anhydrous ethanol, and vacuum-dried for further use.

Maleimide-activated PLNPs-NH_2_ were prepared using the same method. The resulting maleimide-activated PLNPs-NH_2_ were separated by centrifugation, rinsed with ultrapure water, and re-dispersed in 3 mL of PBS buffer. The activated PLNPs-NH_2_ were then reacted with cDNA (3 nmol) at room temperature under shaking overnight. The obtained PLNPs-cDNA were collected by centrifugation and washed with PBS buffer to remove unmodified cDNA.

### 2.5. Preparation of Fe_3_O_4_-SEapt@PLNPs-cDNA

PLNPs-cDNA solution (1 mg/mL, 200 μL) was mixed with Fe_3_O_4_-SEapt solution (1.2 mg/mL, 200 μL), followed by the addition of PBS buffer to adjust the total volume to 1 mL. The mixture was incubated with gentle shaking at room temperature for 2 h. The resulting composite probe, Fe_3_O_4_-SEapt@PLNPs-cDNA, was magnetically separated, thoroughly washed with PBS buffer, and subsequently re-dispersed in 1 mL of PBS buffer.

### 2.6. Preparation of Egg Samples

Pasteurized and untreated chicken eggs intended for raw consumption were used as samples for analysis. Firstly, eggshell surface sample was prepared. Eggs were immersed in 100 mL of buffer solution. After sealing, the container was vortexed for 2 min or placed on a horizontal shaker at 200 rpm for 10 min to fully release bacteria from the eggshell surface. The eggs were then removed, and the eggshell wash solution was stored at 4 °C until use. An aliquot of 1 mL was taken for detection. In addition, eggshell content sample was prepared for analysis. The eggshell surface was disinfected with ethanol. After cracking the egg, the whole egg content was transferred to a sterile homogenization bag, and nine volumes of buffer were added. The mixture was homogenized using a paddle blender at 400 rpm for 2 min to obtain a 1:10 homogenate, which was stored at 4 °C until use. An aliquot of 1 mL was taken for detection.

### 2.7. Detection of SE

In PBS buffer, 100 μL of SE standard solutions at different concentration levels or 100 μL of sample solutions were mixed with 100 μL of Fe_3_O_4_-SEapt@PLNPs-cDNA probe solution (0.5 mg/mL). PBS buffer was added to bring the total volume to 1 mL, resulting in final SE concentrations of 10^2^, 10^3^, 10^4^, 10^5^, 10^6^, and 10^7^ CFU/mL. The mixture was incubated with shaking for 30 min. After the reaction, unreacted Fe_3_O_4_-SEapt@PLNPs-cDNA and the captured Fe_3_O_4_-SEapt complexes were removed using a magnet, leaving only the released PLNPs-cDNA in the supernatant. The afterglow emission (PL) intensity of the remaining solution at 700 nm was measured on a fluorescence spectrophotometer in phosphorescence mode, with an excitation wavelength of 254 nm and excitation/emission slit widths of 10 nm. Because the presence of SE competitively displaces PLNPs-cDNA from the composite probe Fe_3_O_4_-SEapt@PLNPs-cDNA, higher SE levels result in more PLNPs-cDNA being released. Therefore, the concentration of SE can be quantified based on changes in the afterglow signal of PLNPs-cDNA.

## 3. Results

### 3.1. Principle of the Method Based on Functionalized Persistent Luminescence

**Figure 1:**
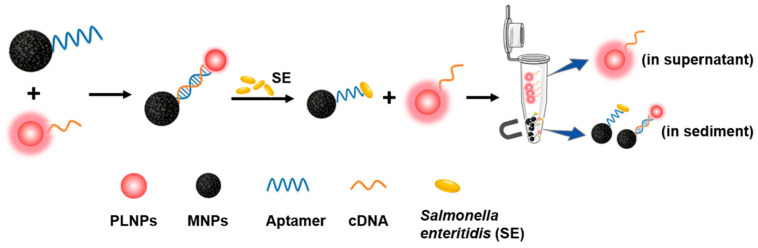
Illustration of the design and principle of the PL aptasensor for *Salmonella enteritidis*.

An optical sensing platform for SE detection was constructed by integrating PLNPs as luminescent probes with aptamer recognition and magnetic separation (Figure 1). PLNPs served as the signal source, as owing to their afterglow emission, autofluorescence interference of food matrix components can be effectively eliminated compared with classical fluorescence-based systems (Figure S1). Using sulfo-SMCC as a heterobifunctional crosslinker, a thiol-terminated SE aptamer was conjugated to Fe_3_O_4_-NH_2_, while cDNA was immobilized on the surface of PLNPs-NH_2_. The composite detection probe, Fe_3_O_4_-SEapt@PLNPs-cDNA, was then assembled via complementary hybridization between the aptamer and cDNA. In the presence of SE, the bacterium binds to the aptamer and competitively displaces the cDNA, leading to the dissociation of PLNPs-cDNA from the Fe_3_O_4_-SEapt@PLNPs-cDNA probe. Excess Fe_3_O_4_-SEapt@PLNPs-cDNA probes and the Fe_3_O_4_-SEapt-SE complexes are subsequently removed by magnetic separation. As a result, only the released PLNPs-cDNA remains in solution, and its afterglow signal shows a positive correlation with the concentration of SE. This optical sensing strategy integrates the unique afterglow property of PLNPs, rapid magnetic separation, and the specific recognition capability of aptamers, thereby improving the sensitivity and selectivity for SE detection.

### 3.2. Characterization of PLNPs

**Figure 2:**
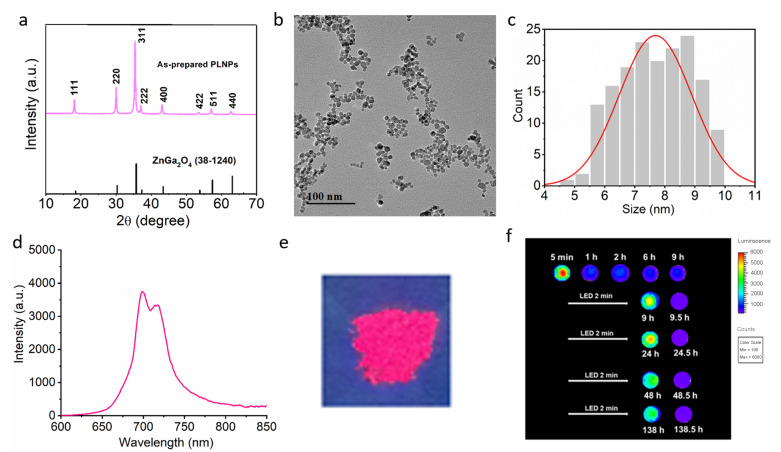
Characterization of PLNPs. (**a**) XRD pattern. (**b**) Transmission electron microscopy (TEM) image. (**c**) Particle size distribution. (**d**) Afterglow emission spectrum of PLNPs. (**e**) Photograph of PLNP powder under UV excitation (254 nm). (**f**) Near-infrared afterglow images of PLNPs (9 h after UV pre-irradiation, followed by re-excitation with red light LED for 2 min).

The XRD pattern indicates that the as-synthesized PLNPs possess the typical spinel structure of zinc gallate (Figure 2a). Transmission electron microscopy (TEM) image shows that the PLNPs are well dispersed with a uniform size distribution and an average diameter of 7.7 ± 1.2 nm (Figure 2b,c). The PLNPs exhibit a near-infrared emission peak at 700 nm arising from the ^2^E-^4^A_2_ transition of Cr^3+^ (Figure 2d). Moreover, because PLNP emission relies on the storage of excitation energy in electron and hole traps, they display pronounced afterglow luminescence in the solid state (Figure 2e). The afterglow performance of the PLNPs was evaluated (Figure 2f). Following pre-irradiation with a 254 nm UV lamp for 5 min and the subsequent termination of external illumination, an afterglow with a duration exceeding 9 h was observed. Furthermore, the PLNPs exhibited excellent reusability, as they could be repeatedly and fully re-excited. These results showed that the PLNPs can continue to emit afterglow luminescence after removal of the excitation light source by utilizing the stored energy, making them well suited for detection applications where fluorescence interference from sample components may be present.

### 3.3. Characteristics of the Probe

**Figure 3:**
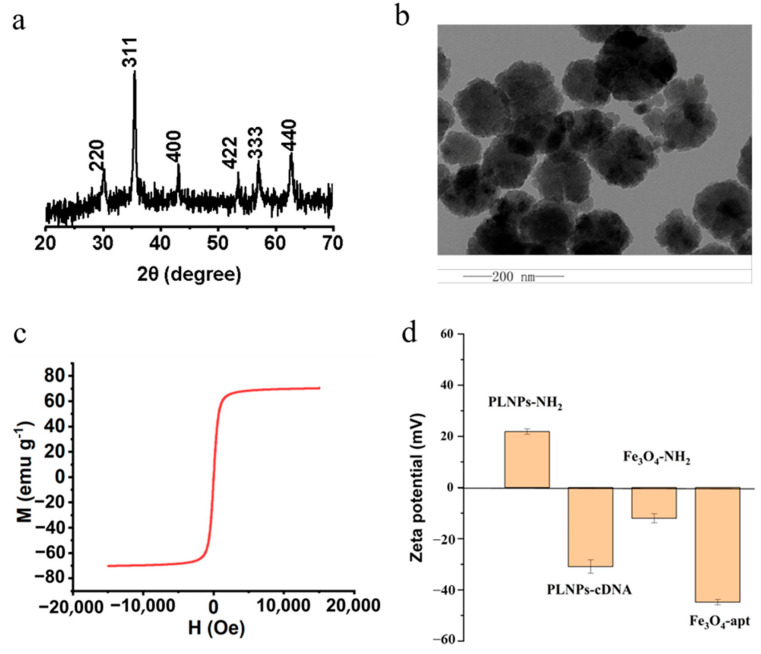
Characterization of Fe_3_O_4_-NH_2_. (**a**) XRD pattern. (**b**) Transmission electron microscopy (TEM) image. (**c**) Magnetic hysteresis loop of Fe_3_O_4_-NH_2_. (**d**) Zeta potential measurements of PLNPs-cDNA and Fe_3_O_4_-SEapt.

Fe_3_O_4_-NH_2_ magnetic nanoparticles were synthesized via a one-step hydrothermal method using 1,6-hexanediamine as an amino ligand and ferric chloride as the iron source. The X-ray diffraction (XRD) pattern shows that the diffraction peaks of the as-prepared are consistent with those of standard Fe_3_O_4_ (JCPDS 82-1533), without any additional impurity peaks, indicating a well-crystallized spinel structure (Figure 3a). TEM images reveal that Fe_3_O_4_-NH_2_ is uniformly dispersed and consists of spherical nanoparticles with a size of approximately 100 nm (Figure 3b). The magnetic properties of Fe_3_O_4_-NH_2_ were characterized (Figure 3c). As shown by the hysteresis loop, Fe_3_O_4_-NH_2_ exhibits a saturation magnetization of 71.2 emu/g, suggesting strong magnetism that is beneficial for subsequent analytical detection. In addition, the preparations of PLNPs-cDNA and Fe_3_O_4_-SEapt were also characterized and validated. Zeta potential results (Figure 3d) revealed that PLNP-NH_2_ initially exhibits a positive zeta potential. Upon modification with the aptamer complementary strand, the surface charge of the nanoparticles is reversed to negative values, which can be attributed to the negatively charged phosphate backbone of the aptamer complementary strand, thereby forming PLNPs-cDNA. Similarly, the zeta potential of Fe_3_O_4_-SEapt becomes −44.8 mV. The UV-vis absorption spectrum (Figure S2) revealed that since cDNA exhibits an absorption peak at 260 nm, the probe PLNPs-cDNA also showed a distinct absorption peak at 260 nm after cDNA modification. Based on the change in the absorption peak at 260 nm of the solution before and after the reaction between cDNA and PLNPs (Figure S3), the loading amount of the cDNA strand on PLNPs was calculated to be 3.47 ± 0.02 μg/mg. Similarly, the modification of SEapt on Fe_3_O_4_ was calculated to be 6.79 ± 0.04 μg mg^−1^ (Figures S4 and S5). The above results indicate the successful functionalization of cDNA and SEapt on PLNPs and Fe_3_O_4_, respectively.

### 3.4. Optimization of Detection Conditions

**Figure 4:**
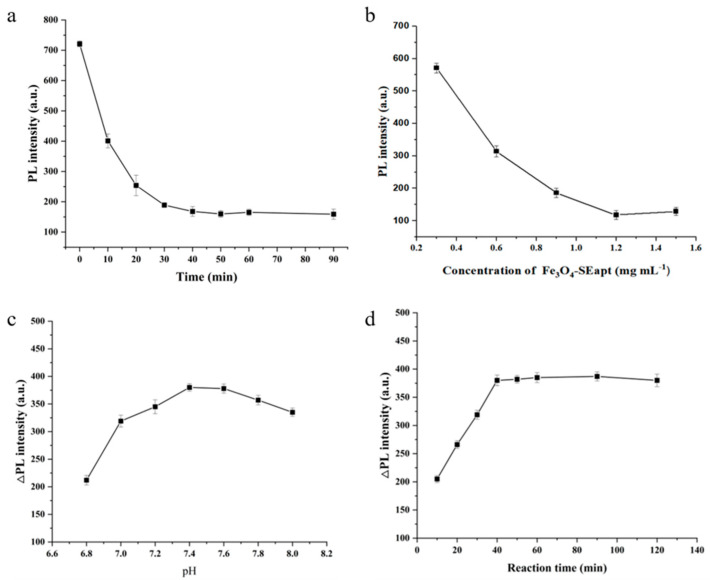
Optimization of detection conditions. (**a**) Hybridization time for composite probe preparation. (**b**) Fe_3_O_4_-SEapt probe concentration. (**c**) Detection pH. (**d**) Detection reaction time (*n* = 3).

With the concentration of PLNPs-cDNA fixed at 1 mg/mL, the effects of hybridization time and Fe_3_O_4_-SEapt concentration on the formation of the composite probe through complementary hybridization between Fe_3_O_4_-SEapt and PLNPs-cDNA were investigated. As more composite probe Fe_3_O_4_-SEapt@PLNPs-cDNA is formed, fewer PLNPs-cDNA remain in the reaction system after magnetic separation, resulting in a weaker afterglow signal. As expected, prolonging the hybridization time led to a gradual decrease in the afterglow intensity of the supernatant after magnetic separation, and the signal reached a plateau after 30 min, indicating that 30 min is sufficient for complete hybridization between Fe_3_O_4_-SEapt and PLNPs-cDNA (Figure 4a). The effect of Fe_3_O_4_-SEapt concentration was then examined. Similarly, with a fixed hybridization time of 30 min, increasing the concentration of Fe_3_O_4_-SEapt resulted in a progressive decrease in the afterglow intensity of the supernatant after magnetic separation, suggesting that a higher Fe_3_O_4_-SEapt concentration increases the collision probability between reactants within the same time frame and thereby promotes the formation of more composite probe (Figure 4b). Considering that an excess of Fe_3_O_4_-SEapt may bind to the target and reduce detection sensitivity, 1.2 mg mL^−1^ Fe_3_O_4_-SEapt was selected, which was sufficient to fully react with PLNPs-cDNA without leaving excessive residual Fe_3_O_4_-SEapt.

To improve the sensitivity of the composite probe toward target recognition, the influence of pH on the detection performance was further investigated, with the target concentration fixed at 1.0 × 10^4^. The afterglow intensity was lower under more acidic or more alkaline conditions than under a slightly alkaline environment (pH 7.2–7.6), which may be attributed to reduced aptamer binding responsiveness toward the target under acidic or alkaline conditions, thereby decreasing the amount of PLNPs-cDNA competitively released into solution (Figure 4c). The effect of reaction time was also evaluated. The afterglow intensity increased rapidly within the first 40 min and then gradually approached a steady state (Figure 4d). To ensure sufficient interaction between SE and Fe_3_O_4_-SEapt@PLNPs-cDNA, a reaction time of 40 min was selected.

### 3.5. Analytical Performance

**Figure 5:**
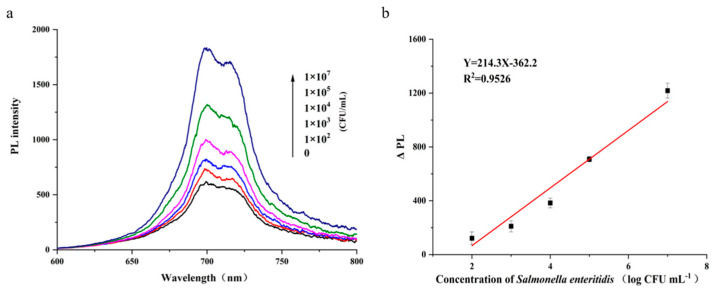
Analytical performance. (**a**) Afterglow signal of the solution after magnetic separation as a function of SE concentration. (**b**) Relationship between ΔPL (the change in afterglow intensity of the supernatant after magnetic separation at 700 nm) and the logarithm of SE concentration (*n* = 3).

Under the optimal detection conditions, a calibration curve for SE was constructed and the analytical performance of the method was evaluated. After magnetic separation, the afterglow intensity increased with increasing SE concentration, indicating that higher bacterial concentrations competitively released more PLNPs-cDNA and thus led to greater recovery of the afterglow signal (Figure 5a). In the concentration range of 1.0 × 10^2^–1.0 × 10^7^ CFU mL^−1^, ΔPL (the change in afterglow intensity at 700 nm induced by SE) exhibited a linear relationship with the logarithm of SE concentration, with a coefficient of determination (R^2^) of 0.9526 and the limit of detection (LOD, 3σ) was 32 CFU mL^−1^ (Figure 5b). The relative standard deviation (RSD) for 11 repeated measurements of 1.0 × 10^3^ CFU mL^−1^ SE was 3.4%.

### 3.6. Selectivity and Specificity

**Figure 6:**
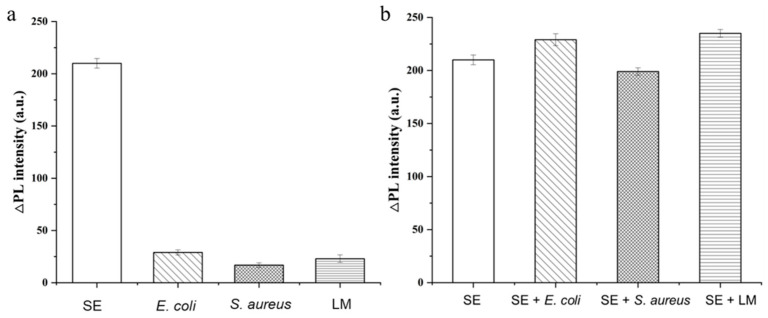
Detection performance. (**a**) Specificity. (**b**) Anti-interference ability (*n* = 3).

To validate the anti-interference capability of the proposed sensing system and its specificity toward SE, spiked samples containing other foodborne bacteria (i.e., *Escherichia coli* (*E. coli*), *Staphylococcus aureus* (*S. aureus*), and *Listeria monocytogenes* (LM)) were analyzed (the concentration of the SE and other foodborne bacteria was 1.0 × 10^4^ CFU mL^−1^). A distinct signal response was observed only in samples containing SE, whereas no appreciable response was detected in solutions containing the other foodborne bacteria, indicating excellent specificity of the sensing system for SE (Figure 6a,b).

In addition, the specificity and anti-interference performance of the system toward SE were further evaluated in the presence of common foodborne bacterial interferents. When the concentration of the other foodborne bacteria was 1.0 × 10^4^ CFU mL^−1^, the recovery for samples spiked with 1.0 × 10^3^ CFU mL^−1^ SE ranged from 98.8% to 102.8%. These results suggest that even in the presence of similar foodborne bacteria at a 10-fold higher concentration, the probe remains essentially unaffected and retains selectivity toward SE (Figure 6b and Table 2).

Similarly, pasteurized and untreated chicken egg samples were selected, and the accuracy of the developed method was validated using the plate-counting method as a reference. The results of SE determination in egg samples by the proposed aptasensor demonstrated that it was detected both on the eggshell surface and in the contents of commercial eggs. In comparison with the conventional plate-counting method, which involves isolating the Salmonella genus from complex microbial floras based on the typical colony morphology on Salmonella-selective media, the proposed method showed bias values ranging from −0.02 to +0.09 log CFU·mL^−1^ for eggshell samples and from −0.11 to +0.05 log CFU·mL^−1^ for egg content samples (Table 3). Although the SEapt employed in our optical sensing platform is specific to SE, the conventional plate-counting method was adopted as the reference method in the analysis of real samples, which does not involve serotyping. Therefore, for real-sample testing, we can only confirm that the developed sensing platform is applicable for the detection of the Salmonella genus. These results demonstrate that, owing to the afterglow luminescence property of PLNPs, the detection signal exhibits strong resistance to interference and provides reliable responses not only in PBS buffer but also in real-sample analysis, without being affected by other bacterial analogues or egg matrix components. This further indicates that, compared with conventional luminescent materials, employing PLNPs as the signal reporting element can afford higher sensitivity and stronger anti-interference capability, supporting the practical relevance and potential applicability of the proposed method.

## 4. Conclusions

In this work, we developed an optical sensing platform for the highly sensitive and specific detection of SE in egg samples by integrating functionalized PLNPs with aptamer-based recognition and magnetic separation. This method takes advantage of the excitation-free and afterglow property of PLNPs to avoid background fluorescence interference, thereby improving the signal-to-noise ratio and sensitivity. The target-recognition capability of the previously reported selective aptamer further enhances the detection specificity, while rapid magnetic separation facilitated by magnetic nanoparticles minimizes interference from other components in the homogeneous solution. The proposed assay exhibited a linear range of 1.0 × 10^2^–1.0 × 10^7^ CFU mL^−1^ with a limit of detection of 32 CFU mL^−1^. This method was successfully applied to the analysis of Salmonella genus in both pasteurized and untreated chicken eggs. Moreover, this strategy can be extended to the detection of other analytes in food samples by replacing the target-specific aptamer. However, such adaptation would require target-dependent optimization and validation.

## Figures and Tables

**Table 1:** The sequences of DNA used in this study.

Name	Sequence (5′-3′)
SEapt	SHCCCGACACGCTGGCGGTCTACGGCTCGTTCTCCCCGG CCC
cDNA	GGGCCGGGGAGAACGAGCCGT

**Table 2:** Recovery of *Salmonella enteritidis* in the presence of other interfering bacteria.

Sample	*Salmonella enteritidis*	*Escherichia coli*	*Staphylococcus aureus*	*Listeria monocytogenes*	Recovery (%, Mean ± *s*, *n* = 3)
1	1.0 × 10^3^ CFU mL^−1^	1.0 × 10^4^ CFU mL^−1^	-	-	101.7 ± 3.1
2	1.0 × 10^3^ CFU mL^−1^	-	1.0 × 10^4^ CFU mL^−1^	-	98.8 ± 4.6
3	1.0 × 10^3^ CFU mL^−1^	-	-	1.0 × 10^4^ CFU mL^−1^	102.8 ± 2.1

-: no addition.

**Table 3:** Determination of Salmonella genus in egg samples (*n* = 3).

Source	Sample	Plate Counting (log CFU mL^−1^)	Detection Concentration (log CFU mL^−1^)	Bias (log CFU mL^−1^)
Untreated chicken egg samples (eggshell)	Sample 1	2.35 ± 0.030	2.33 ± 0.034	−0.02
	Sample 2	2.37 ± 0.014	2.42 ± 0.012	+0.05
	Sample 3	2.43 ± 0.049	2.52 ± 0.036	+0.09
Pasteurized egg (eggshell)	Sample 1	ND	ND	NC
	Sample 2	ND	ND	NC
	Sample 3	ND	ND	NC
Untreated chicken egg samples (egg contents)	Sample 1	2.45 ± 0.031	2.34 ± 0.023	−0.11
	Sample 2	2.40 ± 0.031	2.41 ± 0.019	+0.01
	Sample 3	2.47 ± 0.019	2.52 ± 0.033	+0.05
Pasteurized egg (egg contents)	Sample 1	ND	ND	NC
	Sample 2	ND	ND	NC
	Sample 3	ND	ND	NC

ND: not detected. NC: not calculated. Bias (log CFU mL^−1^): Detection concentration − Plate-counting result.

## Data Availability

The original contributions presented in the study are included in the article/Supplementary Materials. Further inquiries can be directed to the corresponding authors.

## Supplementary Materials

The following supporting information can be downloaded at: https://www.mdpi.com/article/10.3390/foods15081273/s1, Figure S1. emission spectrum of egg samples in the fluorescence mode and phosphorescence mode (under 254 nm excitation); Figure S2. UV-vis absorption spectra of PLNPs before (black) and after (red) cDNA conjugation; Figure S3. UV-vis spectra of cDNA before (black) and after (red) coupling reaction; Figure S4. UV-vis absorption spectra of Fe_3_O_4_ before (black) and after (red) SEapt conjugation; Figure S5. UV-vis spectra of SEapt before (black) and after (red) coupling reaction.

## Abbreviations

The following abbreviations are used in this manuscript:

SE	*Salmonella enteritidis*
PLNPs	Persistent luminescent nanoparticles
cDNA	Complementary DNA
SEapt	*Salmonella enteritidis* aptamer
