# Supplementary material for: Functionalized Persistent Luminescence Nanoparticle-Based Magnetic Separation Aptasensor for Autofluorescence-Free Determination of Salmonella enteritidis
Source: Foods. 2026 Apr 8;15(8):1273. doi: 10.3390/foods15081273 (PMC13114931; doi:10.3390/foods15081273)
Supplement: Supplementary file 1 [file foods-15-01273-s001.zip › foods-4220468-supplementary.pdf]

Supplementary Materials

Figure S1. Emission spectrum of egg samples in the fluorescence mode and phosphorescence mode (under 254 nm excitation).

Figure S2. UV-vis absorption spectra of PLNPs before (black) and after (red) cDNA conjugation.

Figure S3. UV-vis spectra of cDNA before (black) and after (red) coupling reaction.

Figure S4. UV-vis absorption spectra of Fe_3O_4 before (black) and after (red) SEapt conjugation.

Figure S5. UV-vis spectra of SEapt before (black) and after (red) coupling reaction.

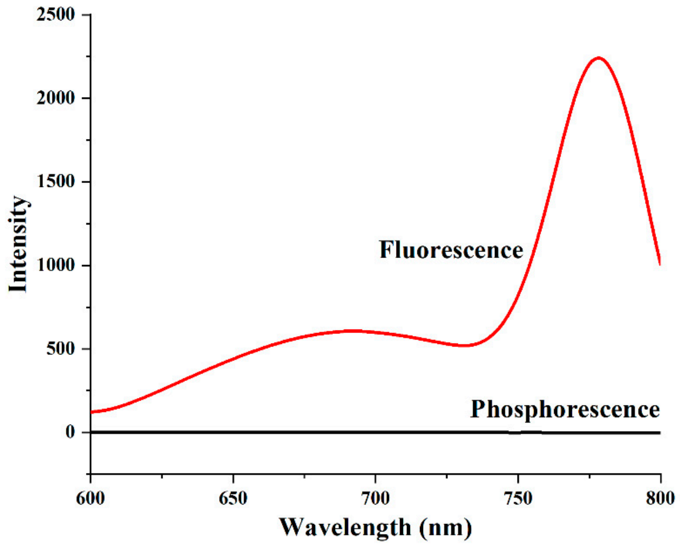

Figure S1. Emission spectrum of egg samples in-the-fluorescence mode and phosphorescence mode (under 254 nm excitation).

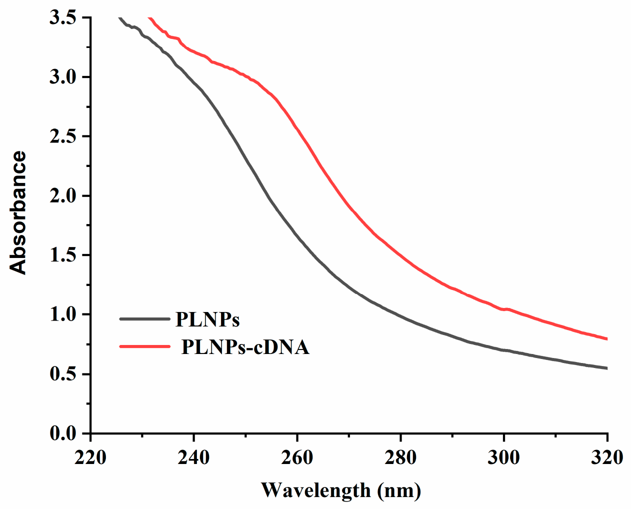

Figure S2. UV-vis absorption spectra of PLNPs before (black) and after (red) cDNA conjugation.

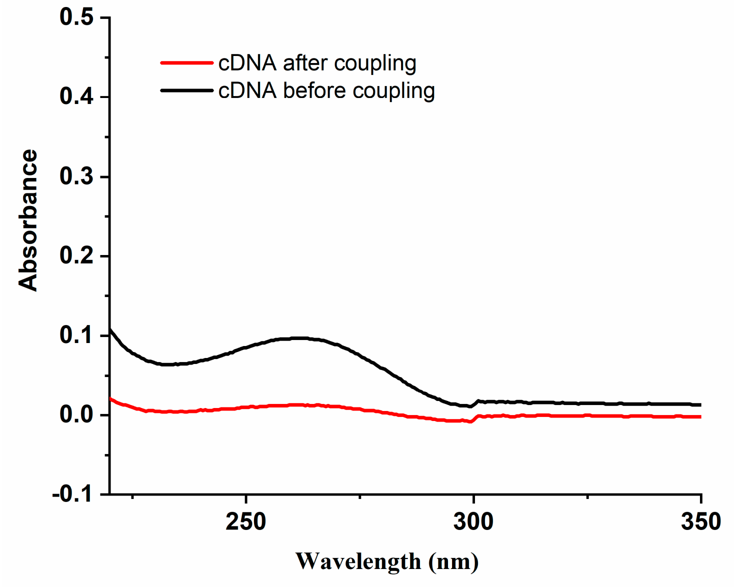

Figure S3. UV-vis spectra of cDNA before (black) and after (red) coupling reaction.

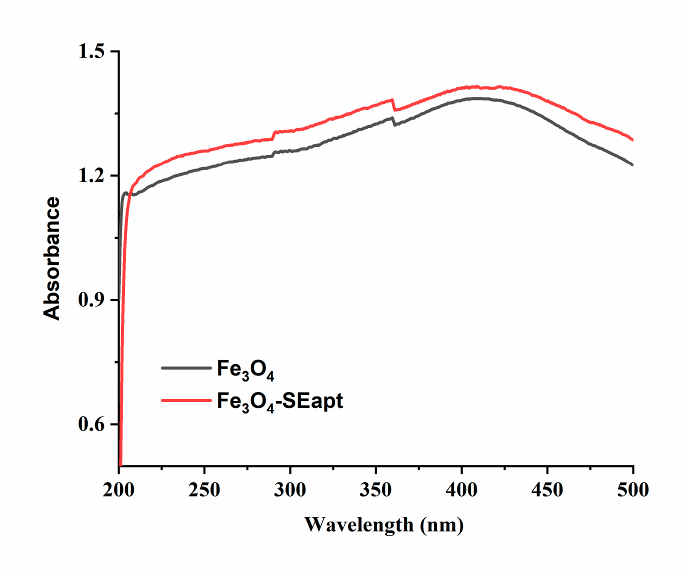

Figure S4. UV-vis absorption spectra of Fe_3O_4 before (black) and after (red) SEapt conjugation.

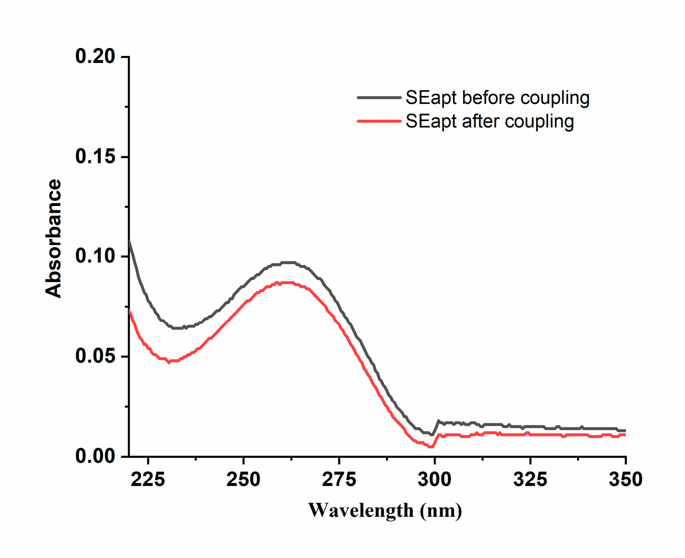

Figure S5. UV-vis spectra of SEapt before (black) and after (red) coupling reaction.
